# Dark current reduction in microjunction-based double electron barrier type-II InAs/InAsSb superlattice long-wavelength infrared photodetectors

**DOI:** 10.1038/s41598-017-13016-9

**Published:** 2017-10-03

**Authors:** Romain Chevallier, Abbas Haddadi, Manijeh Razeghi

**Affiliations:** Center for Quantum Devices, Department of Electrical Engineering and Computer Science, Northwestern University, Evanston, 2220 Campus Drive, RM 4051, Evanston, IL 60208-0893 USA

## Abstract

Microjunction InAs/InAs_1−x_Sb_x_ type-II superlattice-based long-wavelength infrared photodetectors with reduced dark current density were demonstrated. A double electron barrier design was employed to reduce both bulk and surface dark currents. The photodetectors exhibited low surface leakage after passivation with SiO_2_, allowing the use of very small size features without degradation of the dark current. Fabricating microjunction photodetectors (25 × 25 µm^2^ diodes with 10 × 10 µm^2^ microjunctions) in combination with the double electron barrier design results in a dark current density of 6.3 × 10^−6^ A/cm^2^ at 77 K. The device has an 8 µm cut-off wavelength at 77 K and exhibits a quantum efficiency of 31% for a 2 µm-thick absorption region, which results in a specific detectivity value of 1.2 × 10^12^ cm·Hz^1/2^/W.

## Introduction

After InAs/Ga(In)Sb type-II superlattices (T2SLs) were devised by Sai-Halasz *et al*.^1^ and used by Sakaki *et al*. (ref.^2^) to make infrared photodetectors, they have been optimized^3,4^ and have started to challenge mercury cadmium telluride (MCT), which is the state-of-the-art infrared detection technology. Type-II superlattices have been theorized to have multiple advantages over MCT to make infrared photodetectors. MCT compounds are toxic, relatively costly, and can be inhomogeneous across large wafers as opposed to T2SL photodetectors^5^. Additionally, the valence and conduction energy bands can be separately engineered thanks to the flexibility of superlattice structures. This is done by changing the thicknesses of InAs and Ga(In)Sb layers. AlSb layers can also be introduced to further enhance this tunability^6,7^, which allows a bandgap range from 1.5 μm to semi-metals^8,9^.

Nowadays, dark current reduction in T2SL photodetectors is one of the main challenges to increase the specific detectivity (D*), which is the main figure of merit for photodetectors that combines both optical and electrical performance. These photodetectors still suffer from higher dark current density compare to their MCT-based counterparts. The main limiting dark current mechanisms of long-wavelength infrared (LWIR) T2SL photodetectors are the surface leakage and the generation-recombination (G-R) currents^3,10–15^. The G-R current can be described as follows:

1 $${{\boldsymbol{I}}}_{{\boldsymbol{G}}-{\boldsymbol{R}}}({\boldsymbol{V}})=\frac{{\boldsymbol{A}}\ast {\boldsymbol{W}}({\boldsymbol{V}})\ast {\boldsymbol{q}}\ast {{\boldsymbol{n}}}_{{\boldsymbol{i}}}}{2\ast {{\boldsymbol{\tau }}}_{0}}$$

2 $${{\boldsymbol{n}}}_{{\boldsymbol{i}}}=\sqrt{{{\boldsymbol{N}}}_{{\boldsymbol{c}}}\ast {{\boldsymbol{N}}}_{{\boldsymbol{v}}}}\ast {\bf{e}}{\bf{x}}{\bf{p}}(\frac{-{{\boldsymbol{E}}}_{{\boldsymbol{g}}}}{2\ast {{\boldsymbol{k}}}_{{\boldsymbol{B}}}\ast {\boldsymbol{T}}})$$

where *A* is the electrical area of the photodetector, *W(V)* is the depletion region width under a given applied bias voltage (*V*), *n*_*i*_ is the intrinsic carrier concentration, *τ*_0_ is the carrier lifetime, *E*_*g*_ is the bandgap energy of the depletion region, and *N*_*c*_ and *N*_*v*_ are the density of states in the conduction and valence bands, respectively.

The G-R current inversely depends on the carrier lifetime (*τ*_0_). InAs/InAs_1−x_Sb_x_ T2SLs have recently proven to have longer carrier lifetimes than InAs/Ga(In)Sb T2SLs^16^, because the presence of GaSb related native defects inside the InAs/Ga(In)Sb superlattice creates Shockley-Read-Hall recombination centers. Therefore, gallium-free T2SLs are beneficial for decreasing the dark current density in T2SL-based photodetectors. Recently, high-performance LWIR photodetectors based on InAs/InAs_1−x_Sb_x_ T2SLs have been demonstrated^17–20^.

Equations (1) and (2) also show that the G-R current exponentially decreases with increasing semiconductor bandgap energy inside the depletion region. To reduce G-R current further, semiconductor heterostructures have been used to shift the depletion region from the narrow-bandgap absorption region to a large bandgap barrier (Refs^3,11,21^). One example of this concept is the nBn device structure, which is able to reduce the G-R current by pushing the depletion region into a large-bandgap electron barrier area^22^.

**Figure 1:**
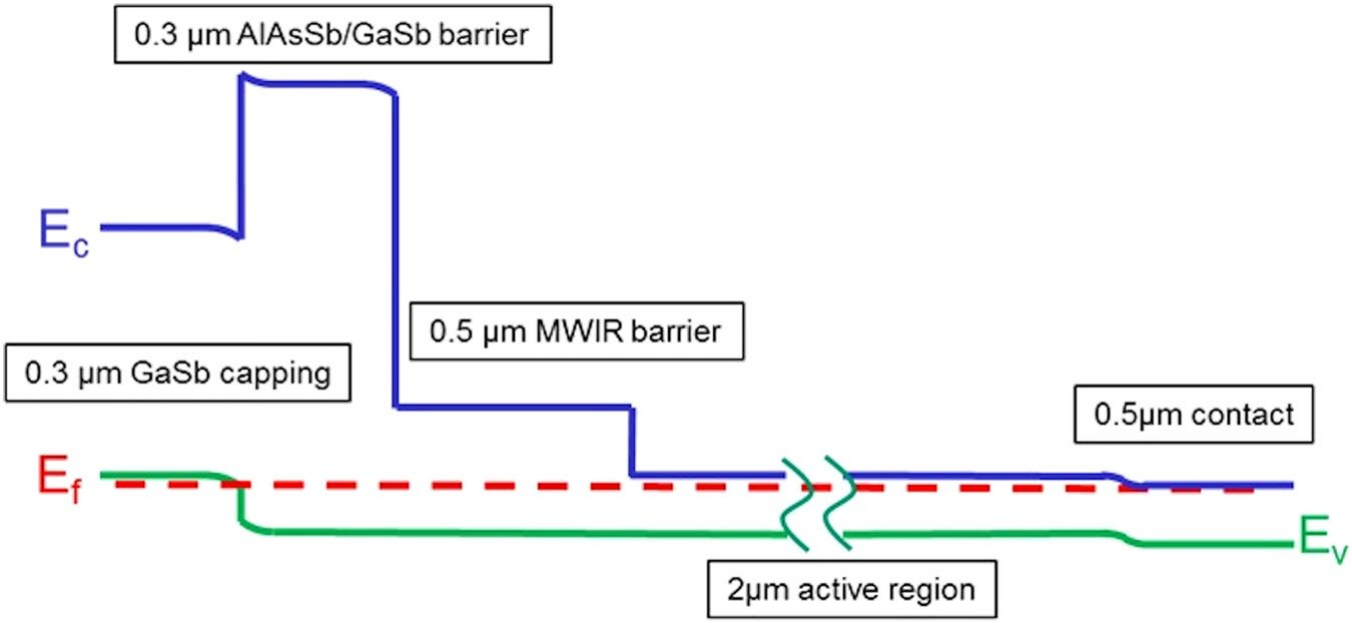
Schematic of the band diagram of C_p_DBn structure.

In this paper, we start with a similar gallium-free InAs/InAsSb nBn device to Haddadi *et al*.^17^. Then we add a second larger-bandgap (E_g_ > 1 eV) AlAsSb/GaSb superlattice barrier^22,23^ after the normal MWIR barrier to create a double electron barrier (Fig. 1). It is similar to the structure for the XDBn, which uses two bulk compound barriers^24^ compared to superlattices in our structure. Using an AlAsSb/GaSb superlattice provides the necessary flexibility to tune the valence band offset while still providing a large energy discontinuity in the conduction band. This additional barrier has a superlattice period of 5/2 monolayers (MLs) of AlAs_0.09_Sb_0.91_/GaSb. It maintains the bias dependency below 200 mV. The top *n*-contact of the original device was then replaced by a 300 nm thick, highly *p*-doped, GaSb capping layer. We call this a *p*-type contact double barrier *n*-type absorption region (C_p_DBn) structure. The addition of this second barrier pushes part of the depletion region into the larger bandgap region, decreasing the G-R current. The larger bandgap barrier also helps suppress surface band-bending and associated surface leakage.

**Figure 2:**
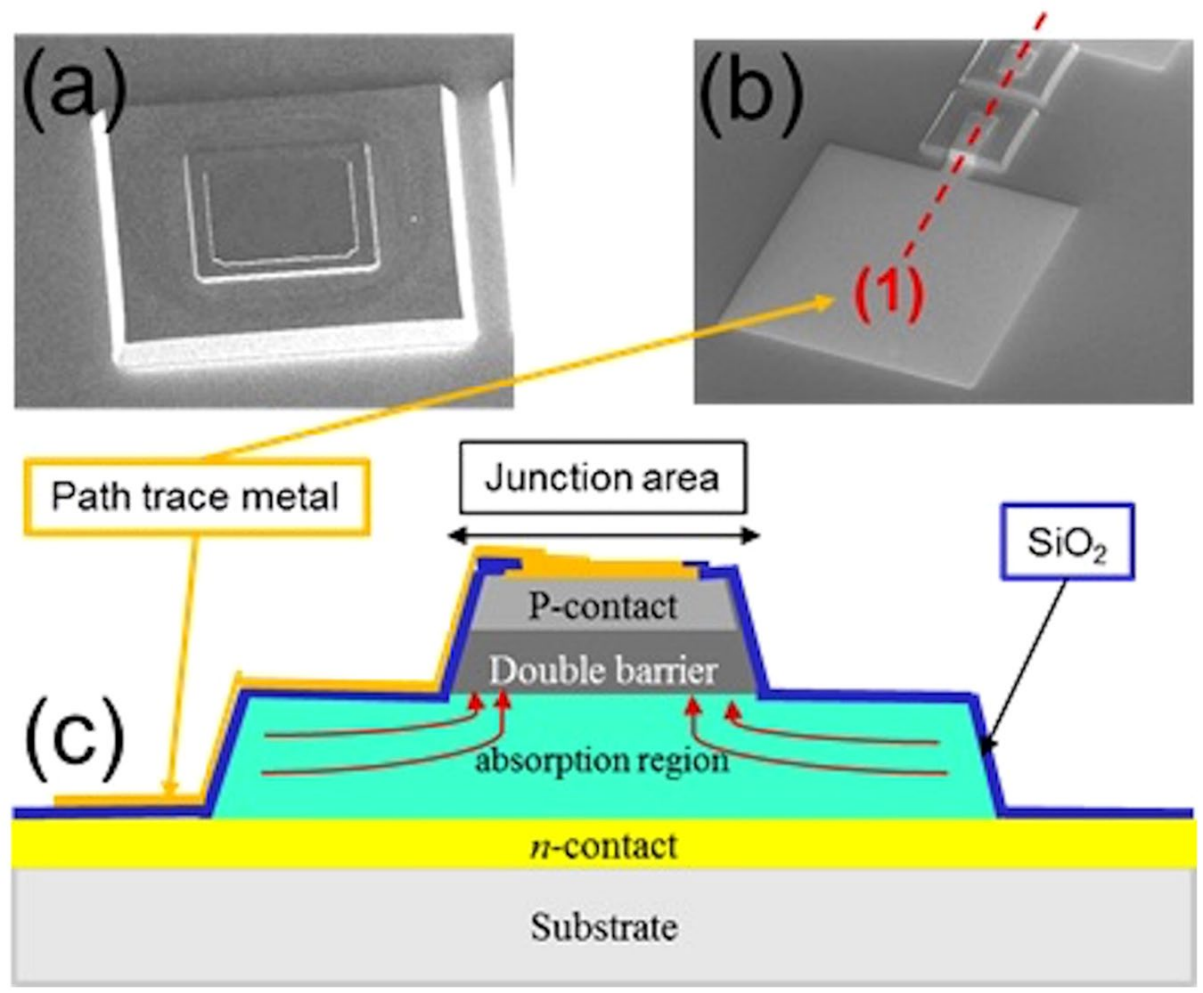
Scanning electron microscope (SEM) picture of microjunction photodiode after (**a**) top and bottom contact metal deposition and after (**b**) path trace metal deposition. (**c**) Cross section schematic of microjunction structure along cross section line (1).

The G-R current is not only proportional to the width remaining in the narrow bandgap portion of the depletion region, but also to the depletion region area (*I*_*G-R*_ *∝ A* * *W(V)*). Due to the reduced surface leakage of our large bandgap double electron barrier, it is now possible to reduce the area of the diode. However, reducing the device area also reduces collection of the optical signal. We can create a microjunction device structure by only etching to the level of the depletion region^25^. The electrical area can be small while the effective optical area is larger. Thus, the G-R current is theoretically decreased by the ratio of the mesa area over the junction area (Fig. 2).

## Results and Discussion

The C_p_DBn structure was grown with a solid-source molecular beam epitaxy (SSMBE) system on an *n*-type Te-doped GaSb wafer. The growth was started by a thin GaSb layer followed by a 500 nm thick InAs_0.91_Sb_0.09_ etch stop layer. It continued with a 500 nm thick bottom *n*-contact, then a 2 μm thick absorption region with a superlattice period comprising 30/10 MLs of InAs and InAs_0.50_Sb_0.50_, respectively. Next a 500 nm thick MWIR barrier was grown, followed by the newly designed 300 nm thick AlAs_0.09_Sb_0.91_/GaSb superlattice barrier, and, finally, a 300 nm thick *p*-doped GaSb capping layer.

**Figure 3:**
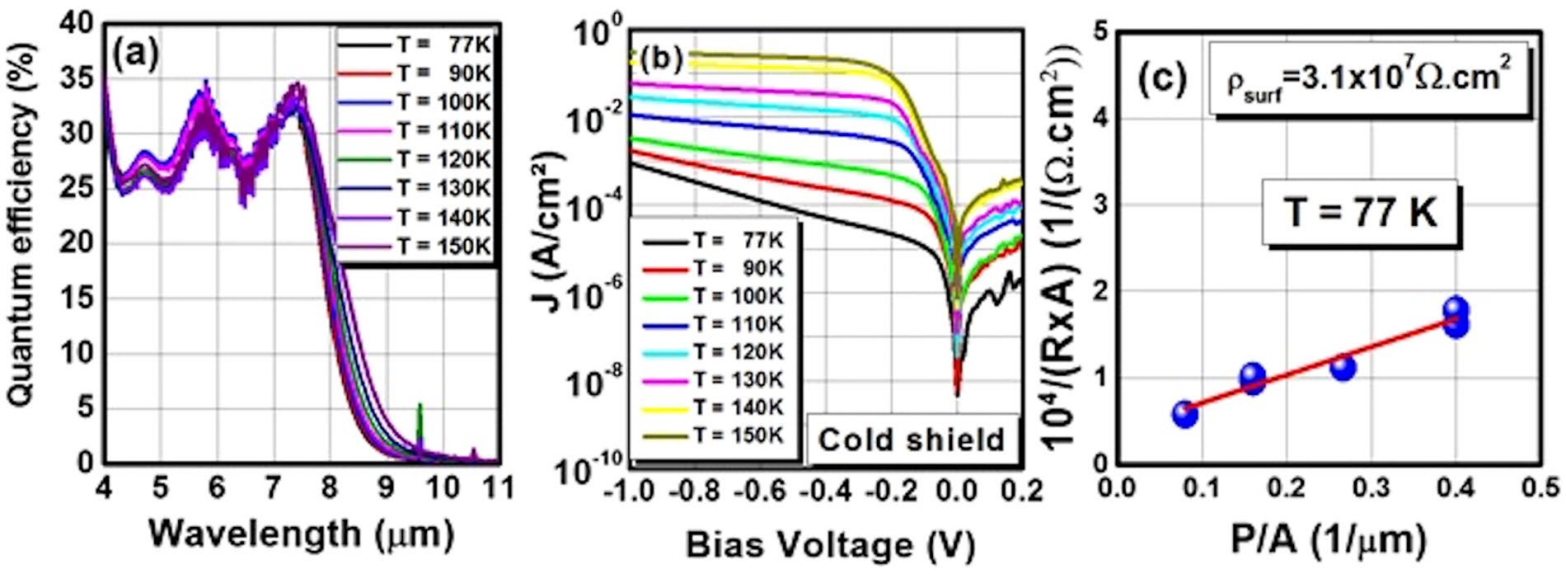
(**a**) Optical performance of unpassivated sample and (**b**) electrical performance of single element **test chip A** 25×25 μm^2^ photodiodes between 77 K and 150 K. (**c**) Resistance area product dependence on P/A for the full-etched passivated single element **test chip A**.

A single element **test chip (A)** with full-etched photodetector areas ranging from 10 × 10 to 400 × 400 µm^2^ was processed using the inductive couple plasma (ICP) dry etching technique. Top and bottom contacts were deposited and the diodes were then passivated with SiO_2_. Windows to the contacts were opened by etching the SiO_2_ layer with a CF_4_ plasma in an electron cyclotron resonance reactive ion etching (ECR-RIE) system. Then metal path traces were deposited on the small-size diodes to wire-bond them, since their contact area is too small for direct wire-bonding. The photodetectors were mounted on a 68-pin chip leadless ceramic chip carrier (LCCC) then wire-bonded and cooled down inside a liquid helium cryostat. An unpassivated single element test chip was also processed to measure the optical performance without influence from the SiO_2_ layer on the optical performance. At 77 K, the single element photodetectors exhibit an 8 μm cut-off wavelength and a peak quantum efficiency (QE) value of ~32% at ~7.3 μm (Fig. 3a) for a 2 µm thick absorption region. **Test chip A** photodetectors have a dark current density (Fig. 3b) of 1.5 × 10^−5^ A/cm^2^ under V_b_ = −140 mV, the bias voltage in which the quantum efficiency spectrum reaches its saturation point. **Test chip A** has a 3.1 × 10^7^ Ω·cm sidewall surface resistivity (Fig. 3c). Having such a high surface resistivity enables the use of small dimensions in the microjunction fabrication without significant surface leakage current degrading the device.

A microjunction **test chip (B)** with various sizes of microjunction was also processed from the same wafer using the same process and passivation, with an additional ICP dry etching step to create the microjunctions. Two parameters in the design of the microjunction must be considered: The ratio of the mesa area over the microjunction area and the distance between the microjunction and the outside collection area. The distance is important because the main mechanism for the lateral collection is diffusion; the diffusion length will limit the lateral collection of the photo-carriers or may require extra bias voltage to collect carriers via a drift mechanism. However, applying additional bias voltage will increase the dark current. Therefore, a compromise will have to be made. With a 25 × 25 µm^2^ mesa, 5 × 5 µm^2^, 10 × 10 µm^2^ and 15 × 15 µm^2^ microjunction photodetectors were processed to study which device design has the best overall performance.

**Figure 4:**
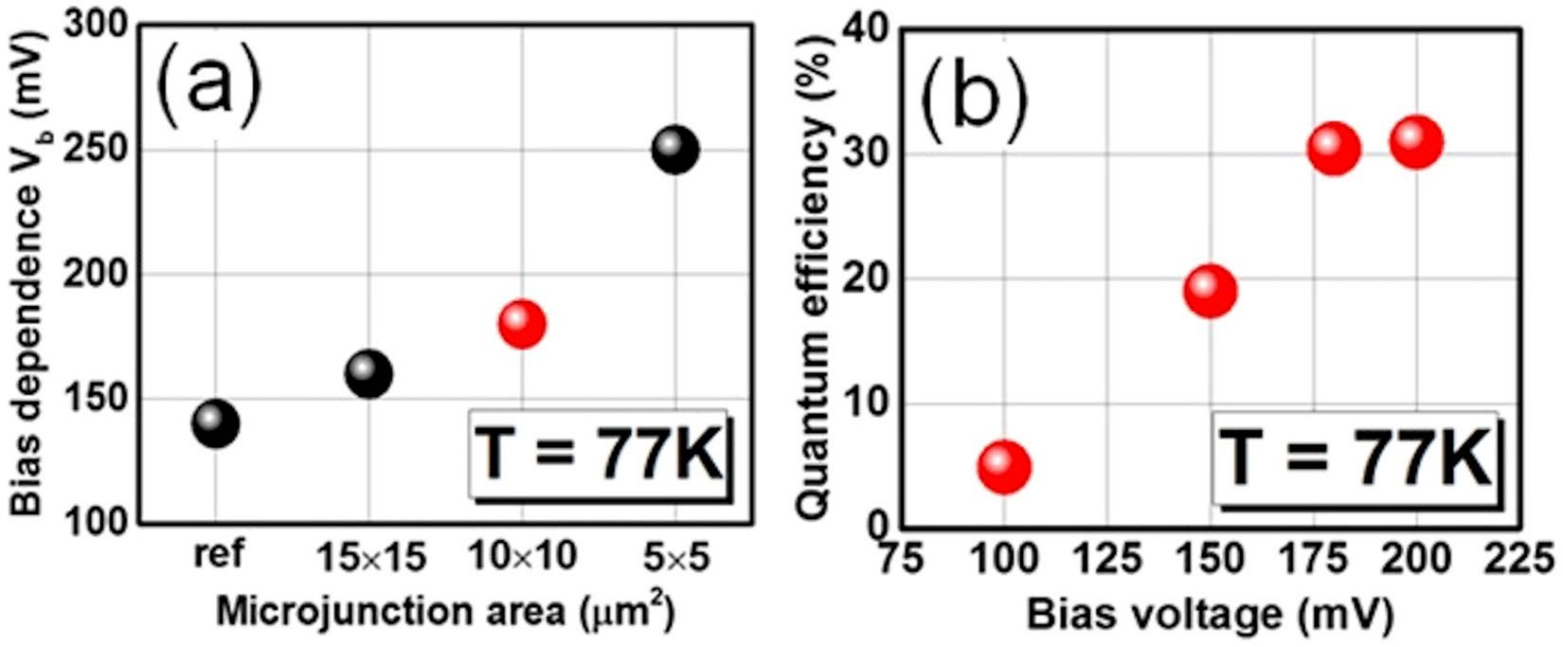
(**a**) Bias dependency of 25 × 25 µm^2^ **test chip A** full-etched mesa photodiode and of **test chip B** 25 × 25 µm^2^ mesa photodiodes with 5 × 5 µm^2^, 10 × 10 µm^2^ and 15 × 15 µm^2^ microjunction. (**b**) Quantum efficiency at 77 K of a 25 × 25 µm^2^ mesa/10 × 10 µm^2^ microjunction photodetector in function of applied bias voltage.

As **test chip B** is passivated with SiO_2_ like **test chip A**, the optical performance of these microjunction photodiodes is compared with **test chip A** to study the bias dependence (Fig. 4) and relative blackbody integrated photo-response. All sizes of microjunction diodes reach full quantum efficiency, however, the bias at which this occurs (bias dependence) increases when the microjunction area becomes smaller (Fig. 4a). The photodetector needs a higher bias voltage to have lateral collection of all photo-carriers.

**Figure 5:**
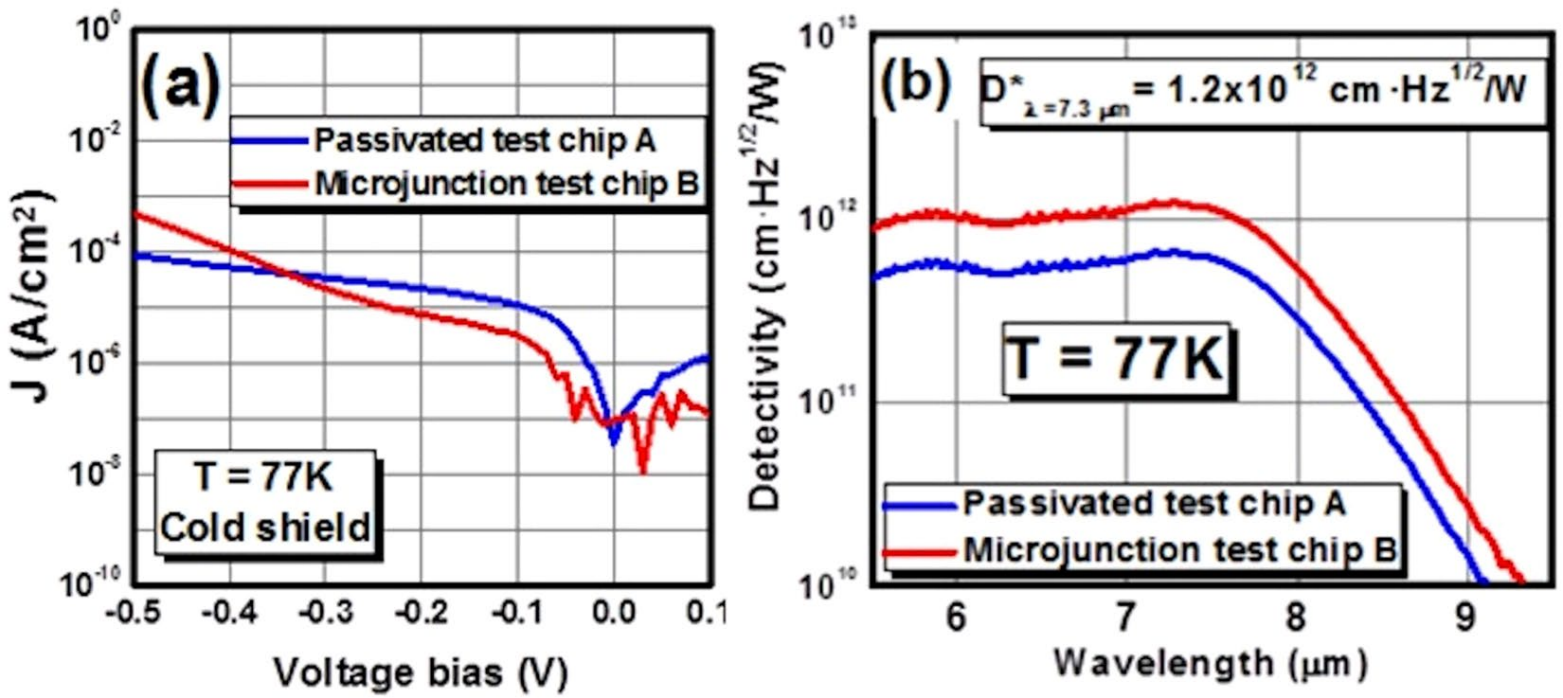
Comparison of (**a**) electrical performance and (**b**) specific detectivity of full-etched 25 × 25 µm^2^ photodiodes from passivated **test chip A** and 25 × 25 µm^2^ mesa/10 × 10 µm^2^ microjunction photodiodes from microjunction **test chip B**.

The electrical performance of the different microjunction photodiodes was measured with a cold shield. As a result, 25 × 25 µm^2^ mesa/10 × 10 µm^2^ microjunction diodes have the best overall performance with a dark current density of 6.3 × 10^−6^ A/cm^2^ (Fig. 5a) at V_b_ = −180 mV quantum efficiency saturation bias (Fig. 4b). Thus, these diodes’ dark current density was decreased 2.5 times compared to **test chip A’s** electrical performance. The dark current of the microjunction photodiode is lower at smaller bias voltages. However, the surface leakage is higher at larger bias voltages because the junction size is smaller. The special detectivity is 1.2 × 10^12^ cm·Hz^1/2^/W for microjunction **test chip B** (Fig. 5b) and is 1.6 higher than **test chip A**.

The 25 × 25 µm^2^ mesa/15 × 15 µm^2^ microjunction diodes showed almost as good performance with 6.5 × 10^−6^ A/cm^2^ at V_b_ = −160 mV, however, the 25 × 25 µm^2^ mesa/5 × 5 µm^2^ microjunction diodes have 1.3 × 10^−5^ A/cm^2^ at V_b_ = −250 mV because the lateral collection requires a significantly larger applied bias voltage.

## Conclusion

In summary, we reported the use of a new *p*-type contact double barrier *n*-type absorption region (C_p_DBn) photodetector structure to improve passivation quality and suppress the G-R current. The double barrier consists of a large bandgap (>1 eV) AlAsSb/GaSb barrier and an undoped MWIR barrier to reduce the G-R current by shifting a portion of the depletion region into this second barrier. The large bandgap barrier suppresses the surface leakage current and the G-R current. This surface leakage suppression allows the use of a microjunction that further improves the electrical performance by reducing the bulk dark current originating from the junction area, especially the G-R current. A study of the bias dependence of optical performance of 5 × 5 µm^2^, 10 × 10 µm^2^ and 15 × 15 µm^2^ area microjunction photodiodes for a 25 × 25 µm^2^ mesa area combined with electrical measurement. The 25 × 25 µm^2^ mesa/10 × 10 µm^2^ microjunction area photodiode structure exhibits a dark current reduction of 2.5 times at the quantum efficiency saturation bias. The structure increases the special detectivity value by 1.6 times to 1.2×10^12^ cm·Hz^1/2^/W.

## Methods

### Growth

To grow the structure, we used a molecular beam epitaxy (MBE) system that is equipped with group III SUMO cells and group V valved crackers. Silicon and beryllium were introduced in the superlattice as *n*-type and *p*-type dopants, respectively. The structure was grown on tellurium (Te)-doped (001) GaSb n-type substrate.

### Fabrication

The processing of single element test chips can be separated in five steps: Mesa isolation, metal contacts deposition, passivation, window opening, and path trace metal deposition. After removing residue from the surface, a standard lithography defines mesa shapes and sizes. The sizes vary from 10 × 10 µm^2^ to 400 × 400 µm^2^. The etching is performed first with an inductive coupled plasma reactive ion etching (ICP-RIE) system with a BCl_3_/Ar gas mixture. After the dry etching, no wet etching is used as it has been proven that dry etching only creates less surface leakage for passivated LWIR samples. A solvent-based cleaning procedure in an ultrasonic bath is then performed to clean the sample thoroughly. Then a second lithography is done to define the top and bottom contacts, which are deposited in an electron beam evaporator and consist of a layer of titanium followed by a layer of gold. This metallization was chosen to create ohmic contacts. Lift-off is then performed and the same cleaning procedure is used to clean the diodes sidewalls. A 1.5 µm thick SiO_2_ layer is deposited on the sample using plasma enhanced chemical vapor deposition (PECVD). Then another lithography is performed to define a window to the contact. An electron cyclotron resonance reactive ion etching (ECR-RIE) system is used with a CF_4_ plasma to etch the SiO_2_ layer. As some of the contact sizes are very small, direct wire bonding is not possible and an additional step is necessary: A path trace metal is deposited to bridge the top contact to an adjacent larger pad for wire bonding.

Microjunction test chips undergo a process similar to the passivated single element test chips. However, the microjunction test chip has an extra etching step: The first etching step defines the diode microjunction and the second etching defines the mesa. The microjunction sidewalls are covered by photoresist during the mesa etching and are therefore protected. Then metal contact deposition, passivation, and window opening are performed in the same way as for the passivated single element test chip. As focal plane arrays typically have pixel areas around 25 × 25 µm^2^, diodes were fabricated with this mesa area and microjunction areas of 5 × 5 µm^2^, 10 × 10 µm^2^ and 15 × 15 µm^2^. Since the top contact sizes are very small, direct wire bonding is not possible. The same path trace metal deposition is used to allow wire bonding.

### Device testing

The processed test chips are mounted onto a 68-pin leadless ceramic chip carrier (LCCC) for electrical and optical characterization. The connections from the top and bottom contacts of the sample to the bond pads of the LCC were made with a gold wire bonder. After bonding, the samples were loaded into liquid helium cryostat that has the capability to measure from 4 K to 300 K. The test chips are connected to a switching matrix that allows connection to a semiconductor parameter analyzer to measure the electrical properties, a fourier transform infrared spectroscopy (FTIR) system to measure the optical response, or a lock-in amplifier to measure the calibrated blackbody integrated response.
